# Temporal Changes in Cortical and Hippocampal Expression of Genes Important for Brain Glucose Metabolism Following Controlled Cortical Impact Injury in Mice

**DOI:** 10.3389/fendo.2017.00231

**Published:** 2017-09-11

**Authors:** June Zhou, Mark P. Burns, Linda Huynh, Sonia Villapol, Daniel D. Taub, Juan M. Saavedra, Marc R. Blackman

**Affiliations:** ^1^Research Service, Washington DC VA Medical Center, Washington, DC, United States; ^2^Department of Biochemistry and Molecular Medicine, George Washington University School of Medicine, Washington, DC, United States; ^3^Department of Neuroscience, Georgetown University School of Medicine, Washington, DC, United States; ^4^Translational Medicine Section, Washington DC VA Medical Center, Washington, DC, United States; ^5^Department of Biochemistry and Molecular and Cell Biology, Georgetown University School of Medicine, Washington, DC, United States; ^6^Department of Pharmacology and Physiology, Georgetown University School of Medicine, Washington, DC, United States; ^7^Department of Medicine George Washington University School of Medicine, Washington, DC, United States; ^8^Department of Medicine, Georgetown University School of Medicine, Washington, DC, United States

**Keywords:** gene expression, glucose metabolism, lactate, hexokinase, GPR81, angiotensin II AT1 receptor, telmisartan, traumatic brain injury

## Abstract

Traumatic brain injury (TBI) causes transient increases and subsequent decreases in brain glucose utilization. The underlying molecular pathways are orchestrated processes and poorly understood. In the current study, we determined temporal changes in cortical and hippocampal expression of genes important for brain glucose/lactate metabolism and the effect of a known neuroprotective drug telmisartan on the expression of these genes after experimental TBI. Adult male C57BL/6J mice (*n* = 6/group) underwent sham or unilateral controlled cortical impact (CCI) injury. Their ipsilateral and contralateral cortex and hippocampus were collected 6 h, 1, 3, 7, 14, 21, and 28 days after injury. Expressions of several genes important for brain glucose utilization were determined by qRT-PCR. In results, (1) mRNA levels of three key enzymes in glucose metabolism [hexo kinase (HK) 1, pyruvate kinase, and pyruvate dehydrogenase (PDH)] were all increased 6 h after injury in the contralateral cortex, followed by decreases at subsequent times in the ipsilateral cortex and hippocampus; (2) capillary glucose transporter Glut-1 mRNA increased, while neuronal glucose transporter Glut-3 mRNA decreased, at various times in the ipsilateral cortex and hippocampus; (3) astrocyte lactate transporter MCT-1 mRNA increased, whereas neuronal lactate transporter MCT-2 mRNA decreased in the ipsilateral cortex and hippocampus; (4) HK2 (an isoform of hexokinase) expression increased at all time points in the ipsilateral cortex and hippocampus. GPR81 (lactate receptor) mRNA increased at various time points in the ipsilateral cortex and hippocampus. These temporal alterations in gene expression corresponded closely to the patterns of impaired brain glucose utilization reported in both TBI patients and experimental TBI rodents. The observed changes in hippocampal gene expression were delayed and prolonged, when compared with those in the cortex. The patterns of alterations were specific to different brain regions and exhibited different recovery periods following TBI. Oral administration of telmisartan (1 mg/kg, for 7 days, *n* = 10 per group) ameliorated cortical or hippocampal mRNA for Glut-1/3, MCT-1/2 and PDH in CCI mice. These data provide molecular evidence for dynamic alteration of multiple critical factors in brain glucose metabolism post-TBI and can inform further research for treating brain metabolic disorders post-TBI.

## Introduction

Glucose is an essential fuel for maintaining cellular functions. In particular, the brain uses glucose and its related intermediate metabolites as energetic substrates to support its functions. Under most normal and pathological conditions, brain glucose utilization is well preserved by complex mechanisms, regardless of peripheral glucose fluctuations.

Following traumatic brain injury (TBI), various brain tissues undergo two types of injuries. The primary injury results from direct mechanical damage to the brain. Subsequently, secondary injury elicits a series of pathophysiological cascade, including edema, increased intracranial pressure, hemorrhage, and decreased cerebral blood flow. Cellular processes during the secondary injury phase involve excessive release of excitatory neurotransmitters, activation of ion channels, mitochondrial dysfunction, hypoxia, and inflammation (1, 2), all leading to degradation of cellular structures, apoptosis (3), and affecting brain glucose requirements and utilization (4, 5). In fact, TBI consistently triggers transient increases and prolonged decreases in brain glucose utilization, as assessed by brain imaging in both TBI patients and experimental animals (6–12).

Our current understanding of molecular processes related effects of TBI on brain glucose utilization is limited. This limitation poses a critical barrier in developing potential therapeutic strategies for TBI management. Among more than 300 currently registered human clinical trials targeting to drug intervention of TBI, there are fewer than 10 trials targeted to normalize brain glucose utilization, and the intervention compounds used in these trials are limited to insulin or glucose/lactate (13). In contrast, several classes of FDA-approved pharmacological agents improve peripheral tissue glucose utilization, with clearly identified mechanisms. A better understanding of molecular mechanisms related to brain glucose utilization after TBI will accelerate investigations of these agents regarding their potential similar effect(s) in the posttraumatic-injured brain.

**Figure 1:**
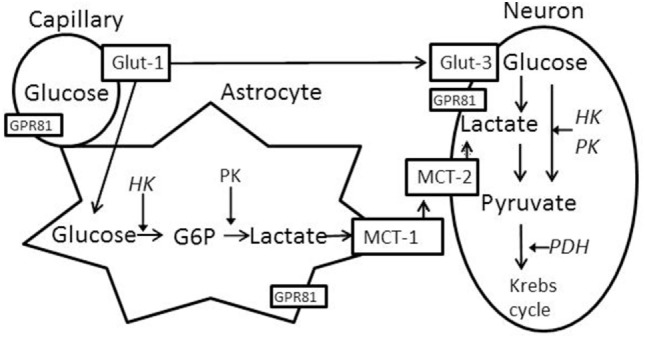
Schematized brain glucose utilization among capillaries, astrocytes and neurons. HK, hexokinase; PK, pyruvate kinase; PDH, pyruvate dehydrogenase; Glut-1, capillary glucose transporters 1; Glut-3, neuronal glucose transport 3; MCT-1, astrocyte monocaboxylate (lactate) transporters 1; MCT-2, neuronal monocaboxylate (lactate) transporters 2. GPR81 (G protein-coupled receptor 81 or hydrocarboxylic acid receptor 1, serve as lactate receptor when lactate is used for signaling molecule).

Brain glucose utilization requires glucose transport and metabolism, as schematized in Figure 1. Glucose transporters (Glut-1 and Glut-3) transport glucose from capillaries to astrocytes and/or neurons (14, 15). Several rate-limiting enzymes play critical roles in glucose metabolism, such as hexokinase 1 (HK1), pyruvate kinase (PK), and pyruvate dehydrogenase (PDH). Additionally, neurons use lactate as an energy substrate to produce ATP (16, 17). Lactate can be produced by glycogenolysis in astrocytes and transported to neurons via monocaboxylate transporters (MCT-1 and MCT-2) (15, 18, 19). HK2 (an isoform of hexokinase) and GPR81 (lactate receptor) are also important in brain glucose metabolism, especially in the injured brain. HK2 expression has been identified in the outer membrane of mitochondria in most brain area and is related to abnormal glycolysis during hypoxia and apoptosis (20). Lactate-activated GPR81 affects several biological pathways in the injured brain, including brain glucose metabolism (21–23). The expression of these enzymes and transporters in various brain tissues is vulnerable to primary and secondary brain injuries.

Several prior reports suggest how various types of TBI influence expression of these enzymes and transporters individually (24–26). In addition, gene array and proteomics studies have examined the expression of large numbers of genes or proteins, including the aforementioned enzymes/transporters, predominantly within a single brain region and/or at a single time point after experimental TBI (27–30). Because brain glucose metabolism is an orchestrated interplay, more fundamental questions remain to be answered. How does TBI simultaneously influence gene expression of multiple key enzymes/transporters that span the gamut of glucose-associated metabolic processes? Also, how does TBI-mediated gene expression vary in diverse brain regions during progression of and recovery from TBI?

In the current studies, we used a controlled cortical impact (CCI) injury mouse model of TBI to investigate: (1) temporal changes in gene expression of selected enzymes and transporters known to be critical for brain glucose utilization, (2) whether a known neuroprotective agent, telmisartan, ameliorates alterations in expression of the abovementioned genes following CCI. Telmisartan is an FDA-approved drug and was originally used for treatment of hypertension, based upon its activity as an angiotensin II AT1 receptor blocker (ARB). Telmisartan also activates peroxisome proliferator-activated receptor gamma (PPARγ), which improves peripheral tissue glucose utilization (31). Neuroprotective effects of telmisartan have recently been reported and are thought to be mediated in part by activating PPARγ (32–35). Because of the complex pathogenesis of TBI, optimized therapies for TBI should target several coexisting pathological mechanisms (36). Considering pleiotropic actions of telmisartan on TBI and glucose metabolism, we examined its effects on expression of genes that regulate to brain glucose utilization in a TBI mouse model.

In study 1, we examined expression of a set of genes related to glucose metabolism at multiple time points to determine if the temporal changes in mRNA expression were related to the temporal changes in brain glucose metabolism after brain injury. These points are chosen to represent early and secondary injuries phases following TBI. In study 2, we examined if gene expression affected by TBI would be ameliorated by telmisartan at a single time point that was selected based on results from study 1. Cortex and hippocampus are the two brain regions that are directly injured or located immediately underneath the injured cortex in our CCI mouse model. Thus, tissues from these two regions contralateral and ipsilateral to the injury were selected for measuring expression of the aforementioned genes.

Our ultimate goal is to develop much needed new therapeutic options for TBI. Data from the current studies will inform subsequent mechanistic studies and broaden our selection of other drugs for their potential role on impaired brain glucose metabolism following TBI.

## Materials and Methods

### Animals and CCI Injury

All animal protocols were approved by the Georgetown University Animal Care and Use Committee and followed National Institutes of Health standards.

Healthy, intact male C57BL/6J mice, aged 8 weeks (Jackson Laboratories, Bar Harbor, ME, USA) were group housed at room temperature of 22 ± 1°C with a 12-h light/dark cycle and *ad libitum* access to food and water. Mice were habituated to the environmental conditions for 7 days. The CCI injury was induced over the parietal cortex as previously described, and was considered to elicit moderate TBI (37). Briefly, the brain is injured by rigid impactor to an intact dura exposed following a craniectomy on the left cortex. The following parameters were used to control impact severity: an impact velocity of 5.25 m/s, a dwell time of 0.1 s, an impact depth of 2.0 mm, and an impactor tip with 3.5 mm diameter. All mice were anesthetized with isoflurane (induction at 4% and maintenance at 2%) evaporated in oxygen and administered through a nose mask. Anesthesia depth was monitored by assessing respiration rate and pedal withdrawal reflexes. The mice were placed on a custom-made stereotaxic frame with a built-in heating bed that maintained body temperature at 37°C. After injury, the incisions were closed with staples, anesthesia was terminated, and the animals were placed in heated cages to maintain normal core temperature for 45 min postinjury. Sham-injured mice underwent the same procedures, including anesthesia, stereotaxic mounting, skin and fascia reflection, and incision closing with staples, but a cranial window was not introduced as we consider this to be part of the primary injury sequence in the CCI model.

In experiment 1, mice were decapitated without isoflurane anesthesia at seven different time points (6 h, 1, 3, 7, 14, 21, and 28 days) after CCI or sham injury (*n* = 6 per group). In experiment 2, mice were divided into vehicle or telmisartan groups after CCI or sham injury (*n* = 10/group, a total of four groups). Telmisartan (Sigma-Aldrich, 1 mg/kg, dissolved in 1% DMSO diluted in distilled water) or vehicle (1% DMSO diluted in distilled water) were administered by oral gavage at a volume of 5 mL/kg. Telmisartan was administrated 1 h post-CCI or sham injury, and once per day for the following 6 days. The experiment was ended 7 days after CCI or sham injury.

For both CCI- and sham-injured mice, the ipsilateral and contralateral parietal cortex and hippocampus were dissected and immediately placed in RNAlater^®^ Solution (Ambion, Thermo Fisher Scientific) and stored at −20°C for total RNA extraction.

The following verifications were performed to ensure accuracy of the real-time PCR TaqMan^®^ method. (1) The same samples were tested with or without DNase treatment for each of the nine target genes (Table 1) to eliminate possible genomic DNA contamination. (2) Standard curves (range from 0.122 to 250 ng/µL total RNA) were assessed for all nine genes to verify the sufficiency and specificity of PCR amplifications for each target gene. The slopes of standard curves were all within a 3.0–3.6 range, and coefficients of determination (*R*^2^) were all larger than 0.99, as required by the TaqMan^®^ real-time PCR assay (3). Because target gene expression must be normalized using endogenous housekeeping genes, it was essential that the endogenous control genes used were not altered by TBI. We tested six different candidate endogenous housekeeping genes (Table 2) in samples collected at different time points after CCI to ensure that the expression level of selected housekeeping gene was stable and not altered by the injury. The variances in expression levels among those samples were calculated for each candidate housekeeping gene (data not shown). The gene with the smallest variance (cyclophilin) was selected for use as the endogenous housekeeping gene. Our results were consistent with a prior publication regarding selection of an endogenous housekeeping gene in mice after experimental TBI (38). To eliminate possible error caused by sample pipetting and differences in RNA quantity and efficiency of reverse transcription for each sample, we established a multiplex q-PCR method for all genes listed in Table 1. Each tested gene was measured in the separate reaction alone, and with cyclophilin, to verify that there was no significant primer dimer formation. The detailed real-time PCR TaqMan^®^ condition was the same as the protocol provided by Applied Biosystems (39). The probes for each target gene and cyclophilin were labeled with FAM and VIC, respectively.

**Table 1:** Brain glucose/lactate utilization related genes.

Gene name	TagMan gene expression ID	Function
Hk1	Mm00439344_m1	Hexokinase 1
PK	Mm00834102_gH	Pyruvate kinase
PDH	Mm00499323_m1	Pyruvate dehydrogenase
Slc2a1	Mm00441480_m1	Glut-1: capillary glucose transporter
Slc2a3	Mm00441483_m1	Glut-3: neuronal glucose transporter
Slc16a1	Mm01306379_m1	MCT-1: astrocyte lactate transport
Slc16a7	Mm00441442_m1	MCT-2: neuronal lactate transporter
GPR81	Mm00558586_s1	lactate receptor
Hk2	Mm00443385_m1	Hexokinase isoform 2

**Table 2:** Housekeeping genes tested.

Gene name	TagMan gene expression ID
Elf1	Mm00468217_m1
Pop4	Mm00481282_m1
Polr2a	Mm00839493_m1
B2m	Mm00437762_m1
Tfrc	Mm00441941_m1
Cyclophilin	Custom designed (40)

After the qRT-PCR method was verified as described above, total RNA was extracted from the dissected tissues using the Trizol reagent following the manufacturer’s protocol, and expression of nine different genes (Table 1) in cortex and hippocampus both ipsilateral and contralateral to the injury were measured by the multiplex q-PCR method. A comparative Ct (ddCt) method was used to determine gene expression levels for each sample.

### Statistical Analysis

Data are presented as mean values ± SEM. SAS 9.2 was used for all analyses. *P* < 0.05 was considerate statistically significant.

For study 1, results were expressed as the percentage change over their corresponding control samples, which were collected at corresponding time points and corresponding (ipsilateral or contralateral) sites from sham-injured mice. Data were analyzed by two-way ANOVA with treatment (Sham vs. TBI) and time post-CCI as two independent factors. Because observed temporal changes varied for different genes and injured sides, the time frames used for calculating TBI effects differed, as specified in the Results section. For those genes that exhibited opposite trends of alterations at 6 h, compared with all other time points following CCI, one-way ANOVA was used to compare TBI and sham group at the 6 h time point only.

For study 2, data were analyzed by *T*-test. Comparison was made within the same injury sides (contralateral or ipsilateral) and the same drug treatment (vehicle or telmisartan).

## Results

### Expression of Genes That Encode Critical Enzymes for Glucose Metabolism

**Figure 2:**
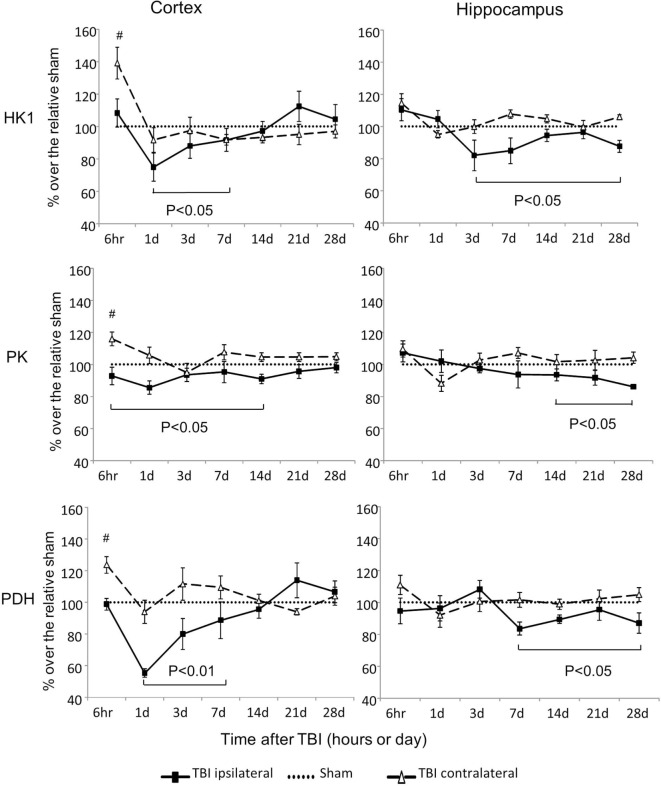
Hexokinase (HK), pyruvate kinase (PK), and pyruvate dehydrogenase (PDH) mRNA levels in the cortex and hippocampus measured at different time points postcontrolled cortical impact (post-CCI). Data are mean ± SEM (*n* = 3–6 per group at each time point for each group). #: *P* < 0.05, contralateral cortex of injured mice compared with the corresponding tissue of sham-injured mice at indicated single time points. Solid brackets indicate the comparison between ipsilateral side of cortex/hippocampus of CCI mice and the corresponding tissue obtained from sham operated mice.

Hexokinase 1, PK, and PDH are three rate-limiting enzymes that play critical roles in glucose metabolism (Figures 1 and 2). mRNA expressions of all three of these enzymes were significantly altered after CCI (Figure 2). However, the alterations differed in regard to brain region, site of injury, and time postinjury. mRNA levels of HK1, PK, and PDH all increased early (6 h) after injury in the contralateral cortex, and all decreased at various subsequent time points in the ipsilateral cortex and hippocampus. Moreover, the observed decreases in the ipsilateral side were kinetically delayed in the hippocampus when compared to those in the cortex. For HK1, the decreases occurred from day 1 to day 7 in the cortex (*P* < 0.05) and from day 3 to day 28 in the hippocampus (*P* < 0.05). For PK, the decreases occurred from 6 h to day 14 in the cortex (*P* < 0.05) and from day 7 to day 28 in the hippocampus (*P* < 0.05). For PDH, the decreases occurred from day 1 to day 7 in the cortex (*P* < 0.01) and from day 7 to day 28 in the hippocampus (*P* < 0.05). In contrast to these ipsilateral decreases, the expression of HK1, PK, and PDH in the contralateral cortex all increased 6 h postinjury (*P* < 0.05 for HK1, PK, and *P* < 0.01 for PDH) and returned to the level of sham controls by day 1 after injury. The only exception was the level of PDH mRNA in the contralateral cortex, which also had transient increase at day 3 postinjury (*P* < 0.05). As expected, in the contralateral hippocampus, none of the three genes exhibited altered expression at any time point following CCI. Together, these results demonstrated that the alterations observed on the ipsilateral side recovered in the cortex within days after injury, but persisted significantly longer in the hippocampus.

### Expression of Genes That Encode Critical Glucose Transporters

**Figure 3:**
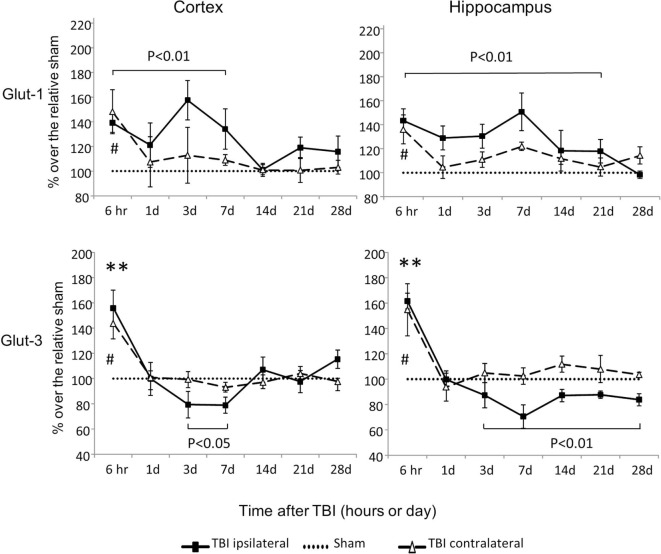
Glut-1and Glut-3 mRNA levels in the cortex and hippocampus measured at different time points postcontrolled cortical impact (post-CCI). Data are mean ± SEM (*n* = 3–6 per group at each time point for each group). #: *P* < 0.05, contralateral cortex/hippocampus of injured mice compared with corresponding tissue obtained from sham-operated mice at indicated single time points. **, *P* < 0.01, ipsilateral cortex/hippocampus of injured mice compared with corresponding tissue obtained from sham-operated mice at indicated single time points. Solid brackets indicate the comparison between the ipsilateral side of cortex/hippocampus from CCI mice and corresponding tissue obtained from sham-operated mice.

Glut-1 is a capillary glucose transporter and Glut-3 is a neuronal glucose transporter. In the contralateral cortex and hippocampus, expression of both Glut-1 and Glut-3 mRNAs increased at 6 h and returned to sham levels by day 1 following injury (*P* < 0.05) (Figure 3). However, in the ipsilateral cortex and hippocampus, CCI-affected Glut-1 and Glut-3 gene expression in an opposite manner. Glut-1 increased from 6 h to day 7 in the cortex (*P* < 0.01) and from 6 h to day 21 in the hippocampus (*P* < 0.01). Glut-3 decreased from day 3 to day 7 in the cortex (*P* < 0.05) and from day 3 to day 28 in the hippocampus (*P* < 0.01), despite transient increases at 6 h (*P* < 0.01 for both cortex and hippocampus). These data suggest that CCI affects the mRNAs of these two glucose transporters in opposite directions, based on their cellular locations. In general, capillary located Glut-1 mRNA increased, while neuronal located Glut-3 decreased.

### Expression of Genes That Encode Critical Lactate Transporters

**Figure 4:**
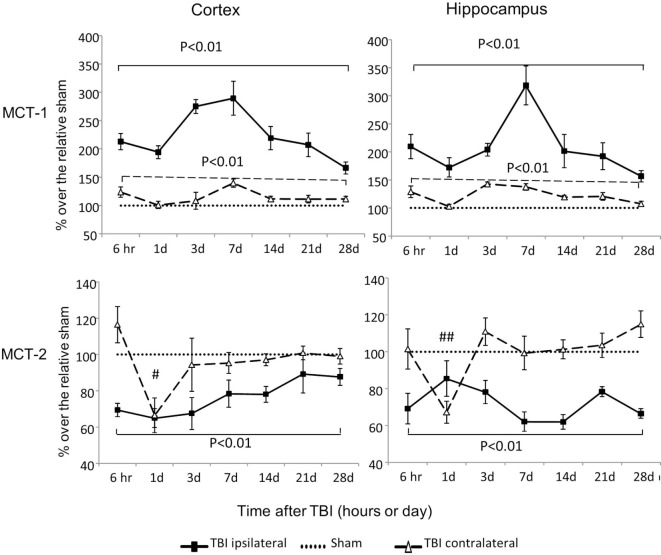
Monocaboxylate transporter (MCT)-1 and MCT-2 mRNA levels in the cortex and hippocampus measured at different time points postcontrolled cortical impact (post-CCI). Data are mean ± SEM (*n* = 3–6 per group at each time point). # or ## *P* < 0.05 or *P* < 0.01, contralateral cortex or hippocampus of injured mice compared with corresponding tissue obtained from sham-operated mice at indicated single time points. Solid brackets indicate the comparison between the ipsilateral side of the cortex or hippocampus from CCI-injured mice and the corresponding tissue obtained from sham-operated mice. Dashed brackets indicate the comparison between contralateral sides of the cortex or hippocampus from CCI-injured mice and the same sides of corresponding tissue obtained from sham-operated mice.

MCT-1 is an astrocyte lactate transporter and MCT-2 is a neuronal lactate transporter. MCT-1 mRNA increased substantially in both cortex and hippocampus, regardless of injury side (*P* < 0.01) (Figure 4). In contrast to these increases in MCT-1, MCT-2 mRNA decreased, and the duration and timing of the decreases were distinctive to injury sides. On the contralateral side, decreases in MCT-2 were only observed at day 1 postinjury for cortex (*P* < 0.05) and hippocampus (*P* < 0.01). On the ipsilateral side, decreases in MCT-2 were persistent from 6 h to day 28 postinjury in both cortex (*P* < 0.01) and hippocampus (*P* < 0.01). We also observed a trend to decreased MCT-2 mRNA returning to sham level in the cortex (time effect *P* < 0.01 by two way ANOVA), but not in the hippocampus. Therefore, similar to glucose transporters, CCI also affected these two lactate transporters’ mRNA in opposite directions based on their cellular locations. In general, mRNA of MCT-1 located in astrocytes increased, while mRNA of MCT-2 located in neurons decreased.

### Expression of Genes That Encode HK2 and Lactate Receptor GPR81

**Figure 5:**
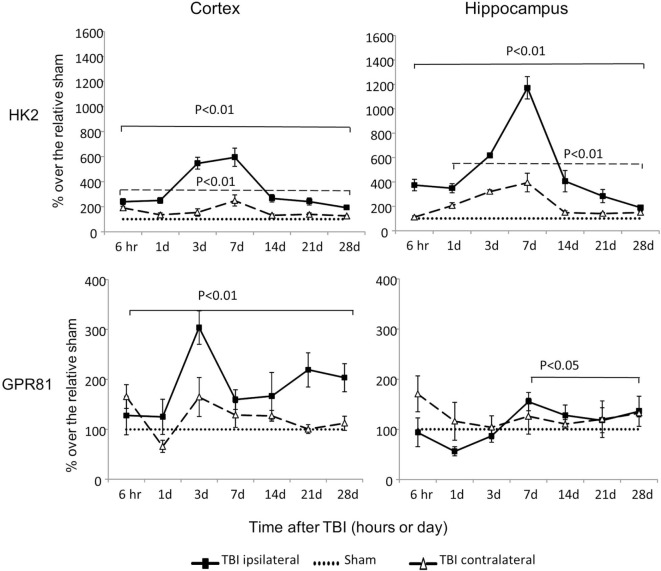
An isoform of hexokinase (HK2) and GPR81 mRNA levels in the cortex and hippocampus measured at different time points postcontrolled cortical impact (post-CCI). Data are mean values ± SEM (*n* = 3–6 per group at each time point). Solid brackets indicate the comparison between ipsilateral sides of the cortex or hippocampus from CCI-injured mice and the corresponding tissue obtained from sham-operated mice. Dashed brackets indicate the comparison between contralateral sides of the cortex or hippocampus from CCI-injured mice and the same side of corresponding tissue obtained from sham-injured mice.

Hexokinase 2 mRNA was dramatically increased at all time points in both cortex and hippocampus, regardless of injury sides (*P* < 0.01), except at 6 h time point on the contralateral side of hippocampus (Figure 5). The increased HK2 expression peaked at day 3 or day 7 postinjury on the ipsilateral cortex and hippocampus, respectively. Notably, among all the genes examined, the ipsilateral increases of HK2 mRNA were the most dramatic: 600% and 1,200% above the sham levels in cortex and hippocampus, respectively.

Controlled cortical impact also increased expression of GPR81 mRNA in an injury side and time specific manner. On the ipsilateral side, GPR81 mRNA levels increased from 6 h to day 28 in the cortex (*P* < 0.01) and from day 7 to day 28 in the hippocampus (*P* < 0.05) post-TBI. On the contralateral side, GPR81 mRNA increased only at day 28 postinjury in the hippocampus (*P* < 0.05), but not in the cortex.

### Telmisartan Selectively Ameliorated CCI-Affected Gene Expression of Glut-1/3, MCT-1/2, and PDH

**Figure 6:**
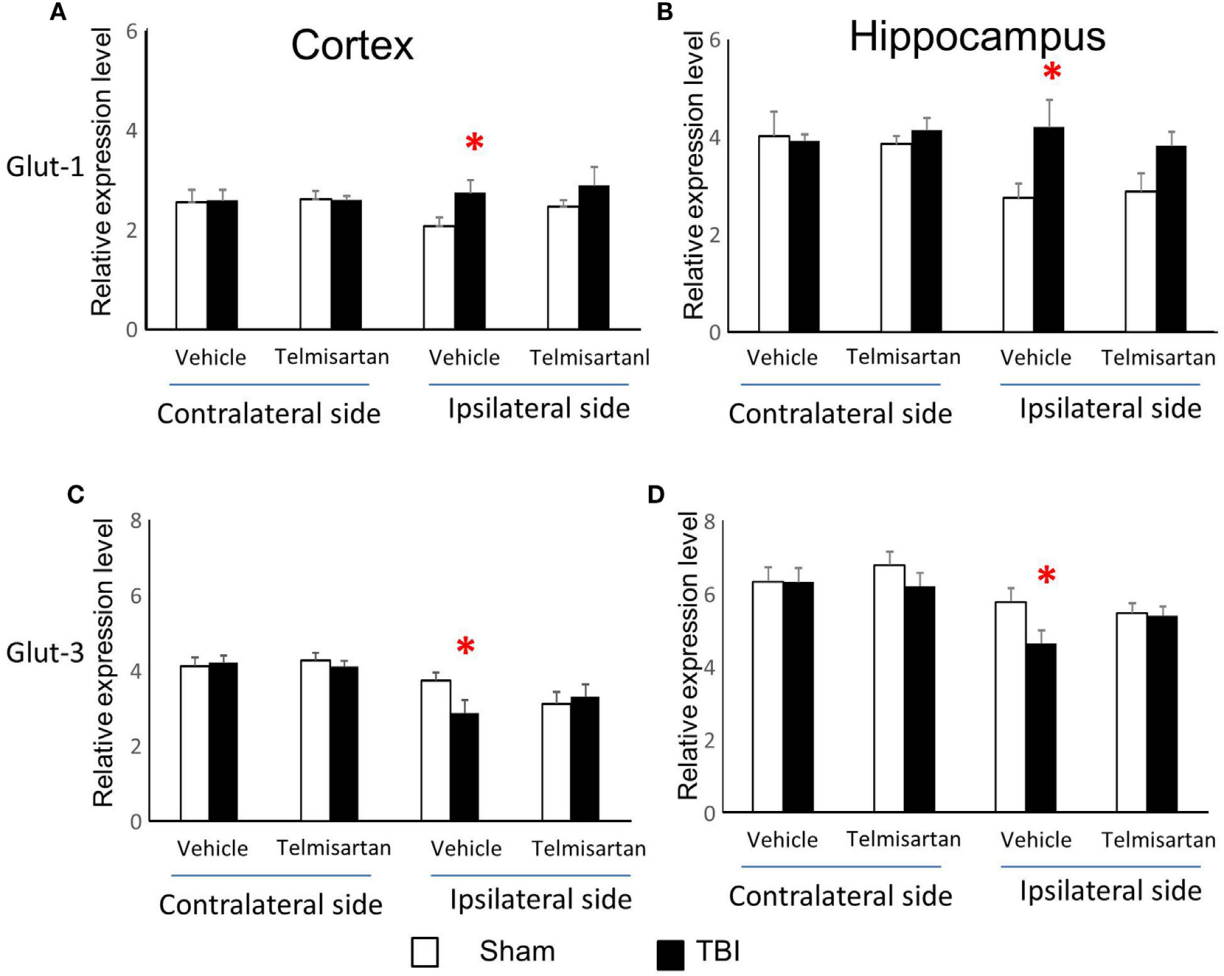
Telmisartan ameliorated controlled cortical impact (CCI)-affected gene expression for Glut-1 and Glut-3. mRNA of capillary glucose transporter Glut-1 **(A,B)** and neuronal glucose transporter Glut-3 **(C,D)** were increased or decreased 7 days after CCI in ipsilateral sides of cortex and hippocampus of mice (*n* = 10/group). Telmisartan (1 mg/kg) revised these changes. *P < 0.05 traumatic brain injury (TBI) vs. Sham.

**Figure 7:**
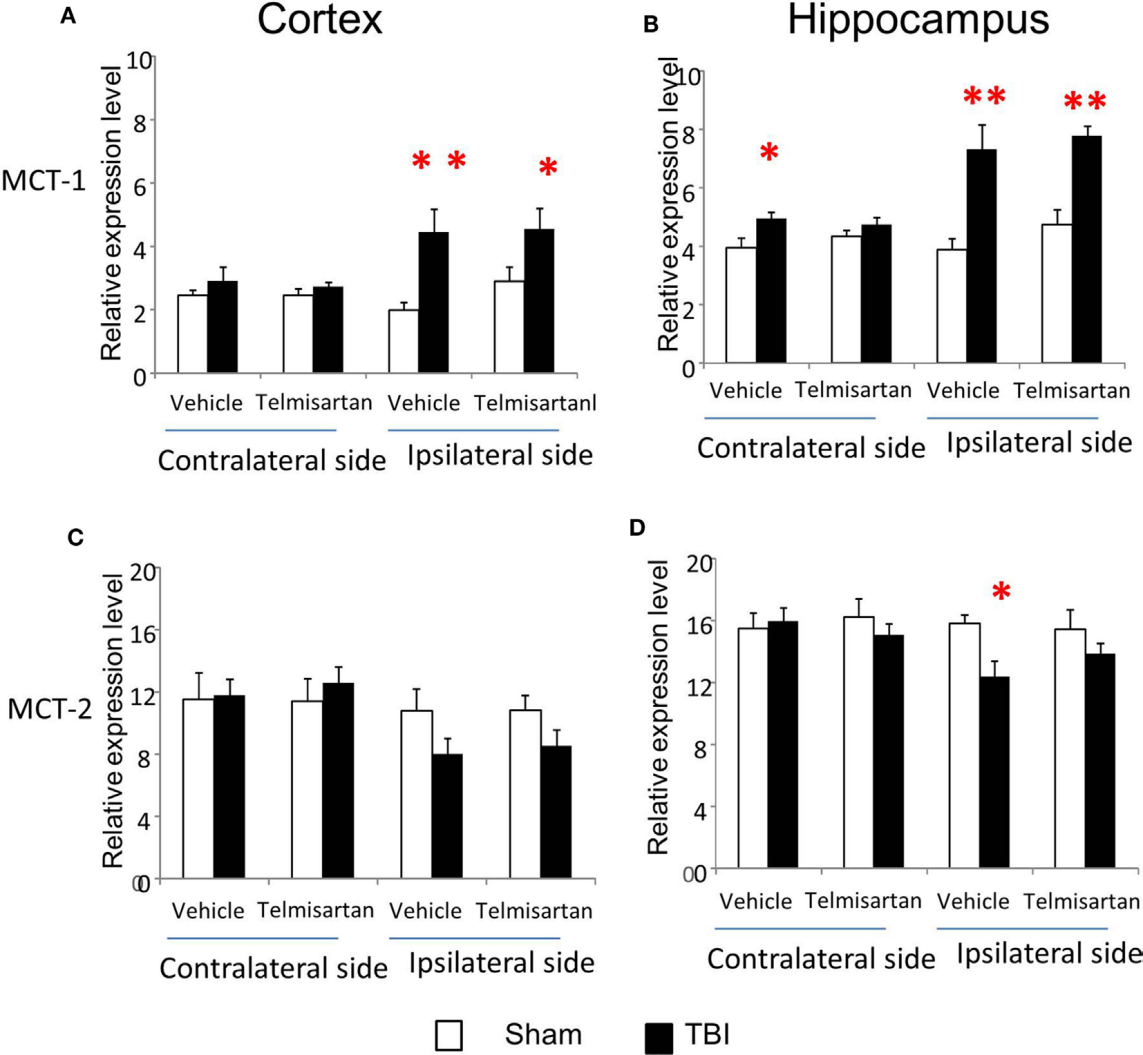
Telmisartan ameliorated controlled cortical impact (CCI)-affected gene expression for MCT-1 and MCT-2. mRNA of astrocyte lactate transporter MCT-1 **(A,B)** and neuronal lactate transporter MCT-2 **(C,D)** were increased or decreased 7 days after CCI in ipsilateral or contralateral sides of cortex and hippocampus of mice (*n* = 10/group). Telmisartan (1 mg/kg) revised these changes. *P < 0.05 traumatic brain injury (TBI) vs. Sham.

From study 1, we found CCI increased gene expression for capillary glucose transporter Glut-1 and astrocyte lactate transporter MCT-1 and decreased gene expression for neuronal glucose transporter Glut-3 and lactate transporter MCT-2 in the ipsilateral side of both cortex and hippocampus (Figures 3 and 4). Such effects were confirmed in study 2 (Figures 3 and 4). However, telmisartan gavage at a dosage of 1 mg/kg for 7 days partially reversed the alterations of Glut-1 and Glut-3 in the ipsilateral cortex and hippocampus (Figure 6). For lactate transporters, telmisartan prevented increased MCT-1 expression in the contralateral, but not ipsilateral, hippocampus (Figure 7B). Compared to MCT-1, the decreased gene expression of MCT-2 was small and were prevented by telmisartan in the ipsilateral hippocampus (Figure 7D).

We also measured HK1, PK, PDH, HK2, and GPR81 gene expression in the contralateral and ipsilateral sides of both cortex and hippocampus. Telmisartan partially reversed the CCI-induced changes in ipsilateral hippocampal PDH mRNA levels, but not other alterations caused by CCI, 7 days postinjury (Table 3).

**Table 3:** Gene expression of HK1, PK, PDH, HK2, and GPR81 measured 7 days post-TBI.

Tissue	Drug	Treat	HK1	PK	PDH	HK2	GPR-81
Contralateral cortex	Vehicle	Sham	1.40 ± 0.06	4.96 ± 0.18	4.75 ± 0.37	8.2 ± 0.81	10.31 ± 1.32
		TBI	1.32 ± 0.05	5.04 ± 0.22	4.62 ± 0.26	14.3 ± 4.3**	15.94 ± 3.29
	Telmisartan	Sham	1.40 ± 0.06	4.52 ± 0.42	4.65 ± 0.25	7.9 ± 0.43	10.53 ± 1.14
		TBI	1.31 ± 0.06	4.59 ± 0.20	4.83 ± 0.31	11.4 ± 0.9**	11.74 ± 1.28

Ipsilateral cortex	Vehicle	Sham	1.54 ± 0.12	3.46 ± 0.26	4.78 ± 0.54	6.5 ± 0.60	7.06 ± 2.02
		TBI	1.35 ± 0.03*	3.77 ± 0.18	4.36 ± 0.34	53.3 ± 11.3**	16.79 ± 1.7**
	Telmisartan	Sham	1.53 ± 0.10	3.72 ± 0.23	4.55 ± 0.35	13.6 ± 6.16	7.92 ± 1.19
		TBI	1.34 ± 0.06*	3.64 ± 0.29	4.83 ± 0.46	34.1 ± 5.2**	15.73 ± 1.9**

Contralateral hippocampus	Vehicle	Sham	1.86 ± 0.10	5.90 ± 0.51	9.79 ± 0.88	13.9 ± 1.57	22.13 ± 3.23
		TBI	1.92 ± 0.10	6.58 ± 0.40	10.36 ± 0.59	32.5 ± 4.4**	27.40 ± 3.84
	Telmisartan	Sham	1.97 ± 0.46	6.04 ± 0.31	9.78 ± 0.70	9.9 ± 1.21	23.30 ± 2.56
		TBI	2.09 ± 0.06	6.03 ± 0.34	10.50 ± 0.52	27.2 ± 2.7**	31.43 ± 5.17

Ipsilateral hippocampus	Vehicle	Sham	1.77 ± 0.10	6.75 ± 0.34	7.77 ± 0.45	11.7 ± 0.56	17.65 ± 2.54
		TBI	1.57 ± 0.07*	6.63 ± 0.39	5.84 ± 0.67*	62.3 ± 10.6**	31.47 ± 6.16*
	Telmisartan	Sham	1.80 ± 0.46	6.25 ± 0.51	7.70 ± 0.34	11.0 ± 0.41	19.40 ± 2.66
		TBI	1.58 ± 0.06*	6.61 ± 0.27	6.36 ± 0.32	75.6 ± 5.4**	27.08 ± 4.57*

*Data are presented as relative ratio of tested gene/cyclophilin and shown as mean ± SE (*n* = 9–10 per group)*.

*P < 0.05 compared with Sham within the same drug treatment in the same brain area.

**P < 0.01 compared with Sham within the same drug treatment in the same brain area.

## Discussion

The temporal alterations in gene expression we observed in these brain areas corresponded closely to transient early increases and subsequent decreases in brain glucose utilization reported in humans and experimental animals following various types of TBI injury (6–12, 41, 42). Six hours after CCI, mRNA levels for two glucose transporters (Glut-1 and Glut-3) and three critical enzymes (HK1, PK, and PDH) were all significantly increased in contralateral brain area. These transient increases might reflect the need for short-term increases in brain glucose utilization immediately following TBI reported previously (6, 7). At various subsequent time points, we observed decreased mRNA expression for HK1, PK, and PDH in the ipsilateral cortex and hippocampus. HK1, PK, and PDH protein levels have been reported to decrease following different types of TBI (29, 43, 44). Thus, our observed decreases in mRNA of three critical enzymes agree with previously reported relative protein changes and the prolonged decreases in brain glucose metabolism.

Interestingly, mRNA for both glucose and lactate transporters changed in opposite directions, depending on the locations of these transporters. Glut-1, predominantly expressed in capillaries (14, 15), increased; whereas neuronal Glut-3 decreased in the ipsilateral cortex and hippocampus. Similarly, lactate transporter MCT-1, predominantly located in astrocytes (15, 18, 19), increased; whereas neuronal lactate transporter MCT-2 decreased in the ipsilateral cortex and hippocampus. These findings suggest that the sustained impairments in brain glucose utilization following TBI may be neuron-specific, despite stimulated expressions of glucose and lactate transporters in capillary and astrocytes. One possible explanation might relate to neuronal susceptibility to death and neuronal loss immediately after injury of the ipsilateral hemisphere. Further research on cell-specific glucose metabolism appears warranted to enhance our understanding of TBI impaired brain glucose utilization.

Changes in hippocampal gene expression were both delayed and prolonged, when compared with those in the cortex. This finding agrees with clinical observations that the hippocampus exhibits prolonged impairments in glucose metabolism when compared with other brain regions, as detected by positron emission tomography (11). For all three key glucose metabolism-related enzymes tested in the current study, their mRNA alterations returned to or toward normal shortly after injury in the cortex. In contrast, alterations of the same three genes persisted for the duration of study in the hippocampus. The underlying molecular mechanisms responsible for these kinetic and tissue-specific changes likely involve multiple factors. One possible factor is permeability of gap junctions, which are intercellular channels connecting the cytoplasm of two cells. These neuronal gap junctions are critically important in the secondary neuronal death following CCI (45). The observation that the gap junctions in the hippocampus are significantly more permeable than those in the cortex (46) might explain why the hippocampus appears to be more vulnerable to harmful molecules resulted from the secondary injury following TBI. Our data also suggest that the delayed and prolonged alteration of these genes in hippocampus may be a cause of the corresponding protracted effects of TBI on memory. Such hypothesis needs to be confirmed by further studies on longer periods after TBI.

The finding of dramatically increased HK2 mRNA expression both contralateral and ipsilateral to the injured cortex and hippocampus was unexpected. HK2 is an isoform of hexokinase. Unlike HK1, the temporal changes of HK2 mRNA expression suggest hypoxia and apoptosis related to abnormal glycolysis (20). Up-regulation of HK2 mRNA is typically associated with aerobic glycolysis rather than oxidative phosphorylation to generate ATP (47), possibly due to the activation of neuroinflammatory responses in microglia and astrocytes (48). Moreover, HK2 expression was associated with poor prognosis and worse overall survival of glioma patients (47, 40, 49). Following CCI, HK2 expression peaked at day 3 or day 7 postinjury and remained two-fold elevated above sham levels at the end of study. These dynamic features and relevance to the recovery process suggest that HK2 mRNA may serve as a biomarker to monitor the progress of injury and recover following TBI. Further studies will determine whether detection of HK2 mRNA in neuron-specific exosomes isolated from peripheral blood can serve as a candidate biomarker for monitoring the progress of TBI.

GPR81 is a G protein-coupled receptor activated by lactate (22, 50, 51). Brain lactate concentrations are significantly increased following TBI (52). The increased lactate can serve both as an alternative brain energetic fuel, and as a signaling molecule affecting several biological pathways in the injured brain (22, 23, 53). Overexpression of GPR81 has been shown to increase cell vulnerability to ischemic injury, whereas inhibition of GPR81 prevented neuronal cell death and reversed brain ischemia-induced apoptosis (53). Thus, GPR81 antagonism has been suggested as a possible therapeutic strategy for the treatment of cerebral ischemia (54). Of note, GPR81 also participates in lactate related neuronal protection initiated by acute organ injury (21, 22, 55). Our findings suggest that use of lactate as a supplementary fuel following brain injury (56, 57) may have complicated consequences. The timing, benefits and harm of such approach requires further investigation.

In brief, CCI-induced alterations in gene expression were specific for the injury side and postinjury time. One possible explanation is that the secondary injury may radiate from initial injury site like a wave to adjacent tissues. Thus, in general, alterations contralateral to the injury were less severe than those ipsilateral to the injury, except at 6 h. Also, alterations in hippocampal gene expression were both delayed and prolonged compared with those in the cortex. These results were of interest because they allow visualization of changes that code most important factors in glucose metabolism.

Telmisartan and other ARBs are strongly neuroprotective in many rodent models of brain disorders, including TBI (35, 58–62) In experimental TBI, telmisartan reduced inflammation and neuronal injury and protected cognition (35, 63–71), effects mediated in part by activating PPARγ (32–35). Telmisartan has also been assessed in patients with Alzheimer’s disease in an NIH approved clinical trial (72). Although telmisartan improved glucose utilization in human peripheral tissues and olfactory tracts (73, 74), its effects on normalizing glucose metabolic-related pathways post-TBI have not been reported. In the current study, we observed that telmisartan improved some, but not all alterations in gene expression 7 days post-TBI. Primarily, CCI-induced changes in mRNAs for glucose/lactate transporters impaired by CCI were partially improved by telmisartan, as well as mRNA for one of three genes encodes critical enzymes related to glucose metabolism. These improvements were only detected when comparison was made between telmisartan-treated Sham vs. TBI groups, not between vehicle-treated TBI vs. telmisartan-treated TBI groups. How these improvements interact with other known neuroprotective pathways require further investigation.

The current findings represent an initial step in our research and need to be complemented, in further studies, with data integrating gene expression with protein levels and glucose utilization. We did not examine the temporal alterations in behavior, biochemistry or histology in this mouse model, as those data have been previously published by our group (37, 58–60). It should also be noted that longitudinal recovery from TBI-induced brain metabolic depression has been related to recovery of behavioral dysfunction assessed by the Morris Water Maze performance (12).

In summary, we measured temporal changes in gene expression of crucial factors that span the interacting pathways associated with glucose metabolism in two key brain regions after CCI in adult mice. The observed temporal alterations in gene expression corresponded closely to temporal alterations in brain glucose utilization reported in TBI patients and experimental animals (6–12). TBI-mediated changes in gene expressions persisted longer in hippocampal vs. cortical tissues and some of those alterations in gene expression were ameliorated by the neuroprotective agent telmisartan. These results will inform subsequent more comprehensive investigations on development of pharmacological interventions that can target the dynamic interplay in brain glucose metabolic disorders following TBI.

## Ethics Statement

All protocols were approved by the Georgetown University Animal Care and Use Committee with National Institutes of Health standards; and Washington DC VA medical Center committee for Research & Development, and Research Safety committee.

## Author Contributions

JZ conceived the experiments, performed the statistical analyses, and wrote the first draft of the manuscript. JZ, MPB, JS, and MRB designed the experiments. JZ, MPB, SV, and LH performed the experiments. JZ, MPB, SV, DT, JS, and MRB contributed to interpretation of data and revisions of the manuscript. All authors contributed to and have approved the final manuscript.

## Conflict of Interest Statement

The authors declare that the research was conducted in the absence of any commercial or financial relationships that could be construed as a potential conflict of interest.
